# Parental occupation and childhood germ cell tumors: a case–control study in Denmark, 1968–2016

**DOI:** 10.1007/s10552-021-01434-0

**Published:** 2021-04-28

**Authors:** Clinton Hall, Johnni Hansen, Jørn Olsen, Di He, Ondine S. von Ehrenstein, Beate Ritz, Julia E. Heck

**Affiliations:** 1 Department of Epidemiology, Fielding School of Public Health, University of California, Los Angeles, Los Angeles, CA, USA; 2 Danish Cancer Society Research Center, Danish Cancer Society, Copenhagen, Denmark; 3 Department of Clinical Epidemiology, Aarhus University Hospital, Aarhus, Denmark; 4 Department of Community Health Sciences, Fielding School of Public Health, University of California, Los Angeles, Los Angeles, CA, USA; 5 College of Health and Public Service, University of North Texas, Denton, TX, USA

**Keywords:** Yolk sac tumor, Teratoma, Social contact, Childhood cancer, Job exposure matrix

## Abstract

**Purpose:**

To examine associations between parental occupation and childhood germ cell tumors (GCTs) in offspring while distinguishing by common histologic subtype (i.e., yolk sac tumor and teratoma).

**Methods:**

This population-based case–control study included childhood GCT cases in Denmark diagnosed 1968–2015 (< 16 years old at diagnosis) and sex and birth year-matched controls. Demographic information and parental employment histories were obtained from Danish registries. Parental occupation was assessed by industry; job-exposure matrices were used to examine specific occupational exposures (i.e., potentially carcinogenic organic solvents and social contact). Conditional multivariable logistic regression models were used to estimate odds ratios (OR) and 95% confidence intervals (CIs).

**Results:**

Overall, 178 childhood GCT cases (50 yolk sac tumors; 65 teratomas) and 4,355 controls were included for analysis. Maternal employment in education during pregnancy was associated with offspring GCTs (OR 2.45, 95% CI 1.23–4.90), especially yolk sac tumors (OR 5.27, 95% CI 1.94–14.28). High levels of both maternal and paternal occupational social contact were also associated with offspring yolk sac tumors across all exposure periods (ORs 2.30–4.63). No signals were observed for paternal occupational solvent exposure, while imprecise associations were estimated for maternal exposure (e.g., dichloromethane exposure during pregnancy, OR 1.51, 95% CI 0.77–2.95).

**Conclusion:**

Our findings suggest that parental occupation is associated with offspring GCTs, with most consistent evidence supporting an association between maternal employment in education or other high social contact jobs and offspring yolk sac tumors.

## Introduction

Childhood germ cell tumors (GCTs) are a rare group of heterogenous neoplasms with largely unknown etiology [1]. In Europe, the incidence rate of GCTs among children ages 0–14 is estimated to be 4.8 per million [2]. In children, the two most common GCT subtypes are yolk sac tumors and teratomas; evidence suggests these cancers have different risk profiles [1, 3], but few observational studies have provided subtype-specific results.

Despite the rarity of epidemiologic studies on childhood GCTs, consistent associations have been observed with Asian/Pacific Islander racial identification, birth defects, and abnormal fetal growth [3, 4]; the latter suggest that prenatal exposures are associated with childhood GCT development. While parental occupational exposures have been examined for several childhood cancers, there are few studies for childhood GCTs. Previously, we observed an association between offspring GCTs and paternal occupational exposure to livestock or animal dust [5]. Associations with parental occupational exposure to chemicals or solvents and plastic or resin fumes have also been suggested [6]. Results were mixed or null for other occupational exposures, such as exhaust fumes, and no study was large enough to stratify by histologic subtype [5–7]. Findings from occupational and environmental studies of parental pesticide exposure and childhood GCT risk are equivocal [8–10].

Other environmental exposure studies have also produced conflicting results for childhood GCTs. Ambient exposure to dichloromethane (methylene chloride) in utero and during the first year of life was associated with childhood GCTs, particularly teratomas, in one case–control study of California children < 6 years old [11]. In other case–control studies by this group, pregnancy exposure to traffic-related air pollution [assessed using the California LINE Source (CALINE4) dispersion model] was particularly associated with teratomas [12], while prenatal exposure to specific traffic-related air toxics (e.g., benzene, toluene, ethyl benzene, and xylenes) was mainly associated with yolk sac tumors [13]. A Spanish case–control study reported a weak association with proximity to urban areas with traffic pollution and childhood GCTs [14]. Other studies have not supported these findings [6, 9, 15].

In this case–control study, which spans several decades, we sought to examine associations between parental occupation, including specific exposures assessed by job-exposure matrices (JEMs), and offspring GCTs. When possible, we separately assessed yolk sac tumors and teratomas to determine whether overall associations with parental occupation differed by histologic subtype.

## Methods

### Data sources and study population

This study was based on a linked database of five national registries in Denmark: The Danish Cancer Registry (data available 1968–2016) [16], the Central Population Registry (1968–2016) [17], the Supplementary Pension Fund (1964–2014) [18], the Medical Birth Register (1973–2016) [19], and the National Patient Register (1977–2016) [20]. Exact linkage of information on a personal level between registries was possible due to the existence of a 10-digit unique personal identifier, including information on birth day and sex, which has been applied to all residents in Denmark since 1968. Information is also stored for the deceased and emigrants.

Childhood GCT cases (< 16 years old at diagnosis) were identified from the Danish Cancer Registry according to the International Classification of Childhood Cancer (ICCC), Version 1 until 2003 and Version 3 thereafter (codes 101–105). Histologic subtypes of GCTs were identified using the International Classification of Diseases for Oncology (ICD-O), Version 1 until 2003 and Version 3 thereafter: yolk sac tumors (ICD-O code 9071) and teratomas (ICD-O codes 9080–9084) were most prevalent in our population. Controls, all of whom were alive and free of cancer at the date of diagnosis of the corresponding case, were randomly selected from the Central Population Registry and frequency matched to cases (1:25) by birth year and sex. Cases and controls had to be born in Denmark to be eligible for this study, and were excluded only if parental occupational exposure history was unavailable for the time periods of interest. In this record-based study, informed consent was not required. Approval for this study was received from the Danish Data Protection Agency and the human subjects’ protection board at the University of California, Los Angeles.

### Exposure assessment

Paternal occupation was assessed during the three months prior to conception and from offspring birth to cancer diagnosis, and maternal occupation was assessed during pregnancy and from offspring birth to cancer diagnosis. Date of conception was calculated using the child’s gestational age as listed in the Medical Birth Registry (see Supplementary File 1 for details). The Supplementary Pension Fund was used to obtain parental employment histories. At its inception in 1964, the Supplementary Pension Fund was compulsory for all salaried employees in Denmark aged 18–66 working at least nine hours per week; in 1978, persons aged 16–17 were additionally included. Students and the self-employed are not covered by the Supplementary Pension Fund [18].

Parental employment was categorized according to a Danish five-digit detailed version of the International Standard Industrial Classification of All Economic Activities [21]. Previously constructed JEMs [22] were used to examine parental occupational exposure to chemicals or solvents previously associated with GCTs: benzene, dichloromethane, gasoline, and toluene [11, 12]. The JEMs employed here were derived from a Finnish template used in the Nordic Occupational Cancer Study; the Danish version of these JEMs were based on measurements in Denmark and expert assessments by JH [23]. The JEMs include industry-specific exposure estimates over four time periods: 1945–1959, 1960–1974, 1975–1984, and 1985+. Binary variables were created to indicate whether a parent had ever held an exposed occupation during the exposure window of interest.

Parental occupational social contact was examined using a JEM that replicated previous work by Kinlen et al. and was updated for the Danish population based on the advice of experts in Danish occupational health [24, 25]. Due to sample size restrictions in this study, occupational social contact exposure was dichotomized (very high/high or medium/low). Occupations with very high social contact included elementary school teachers, daycare workers, and physicians, while occupations with high social contact included other teachers, healthcare professionals, hotel workers, pilots, police, hairdressers, and workers in the transportation industry. Occupations with low social contact included agricultural jobs, and the remainder of occupations were classified as medium social contact.

### Covariate assessment

Child factors assessed included sex, age at diagnosis, place of birth (urban area or rural area/small town), and cryptorchidism diagnosis (yes or no; males only). Age at diagnosis was obtained from the Danish Cancer Registry, and child sex and place of birth were obtained from the Central Population Registry. Diagnosis of cryptorchidism, a correlate of GCT development in males (these tumors are often found in the testes) [26], was obtained from the National Patient Registry, a population-based administrative registry that has collected data from all Danish hospitals since 1977 [20]. The National Patient Registry classified diagnoses according to the International Classification of Diseases, Version 8 (ICD-8) until 1993, with ICD-10 used thereafter; cryptorchidism was identified using ICD-8 codes 752.1x and ICD-10 codes 53.xx. Validation studies have reported that accuracy of information in the National Patient Registry varies by diagnosis, with generally high positive predictive values, but ranging from below 15% to 100%; no specific information on validity of cryptorchidism diagnosis was available [20].

Parental factors assessed included age at offspring birth (≤ 25, 26–30, 31–35, or ≥ 36 years), maternal smoking during pregnancy (yes or no), and family socioeconomic status (SES; high medium–high, medium, medium–low, or low). Parental and gestational information was primarily obtained from the Medical Birth Register, but varied by child’s birth year (described in detail elsewhere [27]). Data on maternal smoking status at first midwife contact were first collected in 1991. Family SES was derived from parental job titles using criteria developed by the Danish National Center for Social Research (high to low: academics or executive managers, middle managers or 3–4 years of further education, other white-collar workers, skilled blue-collar workers, unskilled workers, and unknown or unclassified), as described previously [27].

### Statistical analyses

Conditional logistic regression models were used to estimate odds ratios (OR) and 95% confidence intervals (CI) for associations between parental occupation and offspring GCTs. When sample size allowed, we conducted analyses differentiating the two most common histologic subtypes of GCTs in our population: yolk sac tumors and teratomas. In these stratified analyses, differences between subtypes were assessed via a comparison of point estimates and CIs. For analyses of gasoline and toluene, sensitivity analyses were conducted that restricted exposure to years after 1974 (i.e., after benzene was less commonly used, as these solvents tend to be highly correlated). For analyses of social contact, we performed a sensitivity analysis with further adjustment for exposure to benzene and dichloromethane to account for potential competing exposures. Because the etiology of GCTs is unknown, multivariable models only adjusted for place of birth and parental age (continuous; maternal age for maternal exposures and paternal age for paternal exposures); a sensitivity analysis further adjusting for maternal smoking status during pregnancy was conducted for births 1991 and later. Additionally, because yolk sac tumors and teratomas are typically diagnosed in early childhood and were a secondary focus of this report, sensitivity analyses were conducted subset to prepubertal cases (0–12 years at diagnosis).

Occupational exposures were only reported if there were at least five exposed cases in either parental exposure window. For all analyses, if the number of exposed cases was less than five, risk estimates were not provided and the exposed number was denoted as “ < 5” to comply with statistical uncertainty, and ethics and privacy regulations. The frequency of exposure discordant case–control sets, alongside adjusted ORs and 95% CIs, are provided for the main analysis in Supplementary Tables 1 and 2.

All statistical analyses were conducted using SAS, Version 9.4 (SAS Institute Inc., Cary, NC, USA).

## Results

Initially, 180 GCT cases born in Denmark aged < 16 years old at diagnosis and 4,500 matched controls were identified. After excluding cases and controls without parental occupational exposure history (case *n* = 2; control *n* = 145), the analytic study population consisted of 178 GCT cases (50 yolk sac tumors; 65 teratomas) and 4,355 controls (Table 1). The other common histologic subtypes in this population were germinoma/dysgerminoma (*n* = 33) and embryonal carcinoma (*n* = 12); all other subtypes had fewer than 5 cases. Yolk sac tumors were more prevalent among males and children aged ≤ 5 years at diagnosis. Compared with controls, teratoma cases were more often born in small towns or rural areas, while yolk sac tumor cases were more often born in urban areas. Cryptorchidism was more common among male cases than non-cases. GCT case parents were more frequently older (≥ 36 years old) at date of offspring birth than control parents. Maternal smoking during pregnancy was slightly more prevalent among controls than cases. Family SES was similar between cases and controls.

Paternal employment in professional, scientific, and technical activities during the three months prior to conception was associated with offspring GCTs (Table 2); this association was attenuated when paternal employment from offspring birth to cancer diagnosis was assessed. There were no apparent associations with GCTs and paternal occupational exposure to benzene, dichloromethane, gasoline, or toluene. In subgroup analyses stratified by histologic subtype (Table 3), high or very high paternal occupational social contact during both exposure periods was associated yolk sac tumors. For teratomas, imprecise associations were observed with paternal employment in the food and beverage industry during preconception, and employment in agriculture, forestry, and fishing occupations from offspring birth to cancer diagnosis.

Maternal employment in education during pregnancy was strongly associated with GCTs in offspring, but not employment from offspring birth to cancer diagnosis (Table 4). Elevated point estimates were observed for maternal occupational dichloromethane, toluene, and high/very high social contact exposure during pregnancy and offspring GCTs, but associated confidence intervals were wide and encompassed the null. Maternal employment in two manufacturing sub-industries (i.e., textile, clothing, and leather; and iron, metal works, and foundries) from offspring birth to cancer diagnosis was also associated with GCTs, but effect estimates were imprecise. In analyses that considered histologic subtype (Table 5), maternal employment in education during both exposure windows was strongly associated with yolk sac tumors, as was high/very high maternal occupational social contact. The only maternal exposure associated with teratomas was employment in human health and social work occupations during pregnancy.

Sensitivity analyses adjusting for maternal smoking (births 1991+ only) and those subset to prepubertal cases (0–12 years at diagnosis) did not result in substantial changes to effect estimates, but reduction in sample size precluded the assessment of several occupational exposures (data not shown). For analyses of social contact, sensitivity analyses adjusting for benzene and dichloromethane exposure did not change effect estimates by more than 10% (not shown).

## Discussion

In this nationwide registry-based case–control study spanning several decades, we observed associations between maternal employment in education and other high social contact jobs and offspring GCTs. These findings were stronger in yolk sac tumor cases compared with GCTs overall (there were too few exposed teratoma cases to generate respective risk estimates), an observation which lends support to existing studies that suggest distinct risk profiles for childhood GCT subtypes [1, 3]. Maternal employment in education and JEM classification as high/very high social contact were moderately correlated (pregnancy *r*^2^ = 0.62; offspring birth to cancer diagnosis *r*^2^ = 0.66); and the associations we observed with these exposures suggest infectious and immunologic risk factors for childhood GCTs. One prior study including 451 GCT cases < 6 years identified an imprecise association with maternal Group B streptococcus infection during pregnancy (OR 1.22, 95% CI 0.56–2.65) [3], while another study of 105 malignant GCT cases < 15 years found a strong association with maternal urinary infection during pregnancy (OR 3.1, 95% CI 1.5–6.6), but not with any viral infection (OR 0.4, 95% CI 0.1–1.3) [6]; neither study showed results by histologic subtype. To our knowledge, no other epidemiologic studies have reported on infection and childhood GCTs; however, certain viral infections are thought to be implicated in the pathogenesis of testicular GCTs, which are more common among late adolescent and young adult males [28, 29]. In this study, paternal occupational exposure to high/very high occupational social contact was also associated with yolk sac tumors (but not all GCTs nor teratomas), bolstering the findings we observed with maternal exposure. Notably, parental pre-and postnatal exposure windows for high/very high occupational social contact were fairly correlated (maternal *r*^2^ = 0.59; paternal *r*^2^ = 0.65), but the correlation between maternal and paternal exposure was weak (offspring birth to cancer diagnosis *r*^2^ = 0.16). In all, this is the first study to both investigate and report an association between parental employment in education and other high social contact jobs and offspring GCTs, and additional epidemiologic and mechanistic studies are needed to substantiate these findings.

We also observed associations between offspring GCTs and maternal employment in two manufacturing sub-industries (textile, clothing, and leather; iron, metal works, and foundries) from offspring birth to cancer diagnosis, but case numbers did not allow us to determine if these associations were driven by a specific histologic subtype, or a chance finding. Textile workers are typically exposed to textile-related dusts, including endotoxin; and solvent exposure is common in dyeing and printing operations [30, 31]. One study of parental occupation during the periconception period reported an association between occupational exposure to textile dust and childhood cancer in offspring for a combined category of solid tumors, which included GCTs (maternal exposure OR 1.81, 95% CI 1.28–2.55) [32]. Other studies have reported positive associations between parental occupational dust or solvent exposure and GCTs in offspring [6, 9], but the proportion of these parents employed in the textile industry was unknown. Employment in the iron, metal works, and foundries industry is associated with several exposures including polycyclic aromatic hydrocarbons, silica dust, and metal fumes; cohort studies have identified an increased risk for lung cancer among iron and steel founding workers [33], but more evidence is needed to support an association with offspring GCTs [6, 9].

Although the estimated effect for GCTs and maternal exposure to dichloromethane during pregnancy was imprecise, our point estimate (OR 1.50) was very similar to a recent study of California children (< 6 years old) and ambient dichloromethane exposure in utero (OR 1.52, 95% CI 1.11, 2.08) [11]. In the California study, the association with dichloromethane was stronger for teratomas (OR 2.08, 95% CI 1.38–3.13); in the present study, the small number of exposed cases precluded the derivation of risk estimates for teratomas. Dichloromethane is a solvent which was previously used in aerosols, paint removers, adhesives, and many chemical/industrial processes, and was recently classified as a probable human carcinogen (Group 2A) by the International Agency for Research on Cancer (IARC) [34]. In utero exposure to dichloromethane may disrupt differentiation and migration of early primordial germ cells during neonatal development, leading to carcinogenesis [11, 35].

With the exception of paternal occupational social contact, only paternal employment in professional, scientific, and technical activities during three months preconception was associated with GCTs in this study. This occupational group is varied; in our sample, case fathers were employed in data processing, legal services, accounting, bookkeeping, engineering, land inspection, and architecture. This group may be a proxy for higher SES; however, high/medium–high family SES did not appear to differ between all cases and controls in this study. There was also a suggestion that paternal employment in agriculture, forestry, and fishing occupations from offspring birth to cancer diagnosis was associated with GCTs, particularly teratomas. Of the six exposed teratoma cases, families had paternal employment in crop farming livestock farming, fur farming, and agriculture. A recent study using the same data sources as the present analysis identified an association between paternal occupational exposure to livestock and/or animal dust (derived using a JEM) from offspring birth to cancer diagnosis and GCTs in Danish children < 17 years old (OR 1.82; 95% CI 1.05–3.27), but small numbers did not allow for subtype-specific estimates [5].

In this registry-based study, recall bias and self-selection of participants were not possible and thus could not affect exposure assessment or generate selection bias. However, because we relied on objectively recorded employment histories and JEMs, non-differential exposure misclassification is likely to have occurred. Although the analyses we performed were chosen a priori, we cannot rule out that multiple testing resulted in some chance findings. The social contact JEM used in this study has not been validated for the presumed exposures associated with corresponding occupations [24], though studies have previously found that employment in healthcare and education is associated with higher rates of exposure infectious disease [36, 37]. Still, the occupations considered high/very high social contact in this study are a heterogeneous group with varying levels of exposure to potential chemical and non-chemical carcinogens and we could not account for all competing exposures, though sensitivity analyses adjusting for benzene and dichloromethane exposure made little difference in resulting effect estimates. Although we consider stratification by histologic subtype a strength in the present study, we lacked the sample size to do so for all occupational exposures. While the small number of GCT cases included for analysis yielded low statistical power, this epidemiologic study is still among the largest to examine parental occupational exposures and childhood GCTs. This study was further strengthened by the use of objective records, including a reliable cancer registry that captured cases over an extended period of time in a country with free access to healthcare for all residents.

Additional studies are needed to scrutinize the associations identified in the present report. Research powered to distinguish between histologic subtypes of childhood GCTs would likely be most informative.

## Supplementary Material

1713147_Supp_tab

## Figures and Tables

**Table 1:** Characteristics of germ cell tumor (GCT) cases diagnosed in Denmark aged 0–15 years, and birth year and sex-matched controls, 1968–2016^a^

Characteristic	Controls (n = 4,355)	GCT cases (*n* = 178)		
		All GCTs	Teratomas (*n* = 65)	Yolk sac tumors (*n* = 50)
Child sex				
Male	2,350 (54.0)	96 (54.0)	31 (52.3)	32 (64.0)
Female	2,005 (46.0)	82 (46.0)	34 (47.7)	18 (36.0)
Child age at diagnosis				
Mean ± standard deviation	N/A	7.8 ± 6.3	7.3 ± 6.5	3.6 ± 4.4
Child place of birth				
Urban area	1,492 (34.3)	64 (36.0)	20 (30.8)	22 (44.0)
Rural area or small town	2,863 (65.7)	114 (64.0)	45 (69.2)	28 (56.0)
Child cryptorchidism diagnosis^b^				
Yes	59 (3.7)	6 (9.1)	2 (10.0)	1 (4.2)
No	1,540 (96.3)	60 (90.9)	18 (90.0)	23 (95.8)
Missing	22	0	0	0
Maternal age at birth				
≤ 25 years	1,524 (35.0)	60 (33.7)	19 (29.2)	19 (38.0)
26–30 years	1,622 (37.2)	65 (36.5)	30 (46.2)	16 (32.0)
31–35 years	882 (20.3)	34 (19.1)	8 (12.3)	10 (20.0)
≥ 36 years	327 (7.5)	19 (10.7)	8 (12.3)	5 (10.0)
Paternal age at birth				
≤ 25 years	850 (20.0)	29 (16.3)	6 (9.2)	10 (20.0)
26–30 years	1,586 (36.6)	63 (35.4)	22 (33.9)	19 (38.0)
31–35 years	1,135 (26.2)	49 (27.5)	22 (33.9)	10 (20.0)
≥ 36 years	767 (17.9)	37 (20.8)	15 (23.1)	11 (22.0)
Missing	17	0	0	0
Maternal smoking during pregnancy^c^				
No	1,421 (78.0)	62 (81.6)	23 (88.5)	21 (84.0)
Yes	400 (22.0)	14 (18.4)	3 (11.5)	4 (16.0)
Missing	90	3	3	0
Family socioeconomic status^d^				
High	427 (12.6)	19 (13.9)	9 (18.0)	3 (8.6)
Medium–high	588 (17.4)	23 (16.8)	7 (14.0)	8 (22.9)
Medium	595 (17.6)	24 (17.5)	9 (18.0)	4 (11.4)
Medium–low	1,128 (33.4)	42 (30.7)	13 (26.0)	13 (37.1)
Low	641 (19.0)	29 (21.2)	12 (24.0)	7 (20.0)
Missing	976	41	15	15

a Data presented as, *n* (%) unless otherwise stated

b Data on cryptorchidism diagnoses are reported only for males and births 1977+

c Data on maternal smoking are only reported for births 1991 and later

d Missing data on family socioeconomic status increased over time due to changes in Danish tax law

**Table 2:** Odds ratios (ORs) and 95% confidence intervals (CIs) for associations between paternal occupation/occupational exposures and offspring germ cell tumors, stratified by paternal exposure window, 1968–2016

Occupational exposure	Paternal exposure window					
	Three months preconception to birth			Birth to diagnosis		
	Case *n*/control *n*	OR^a^	aOR^b^ (95% CI)	Case *n* /control *n*	OR^a^	aOR^b^ (95% CI)
Total	148/3,527			163/3,971		
Occupational industry						
Agriculture, forestry, fishing	< 5/120	–	–	11/220	1.25	1.33 (0.70–2.53)
Manufacturing	35/915	0.90	0.90 (0.61–1.33)	56/1,456	0.90	0.93 (0.67–1.31)
Food and beverage industry	9/155	1.40	1.41 (0.70–2.85)	16/320	1.26	1.31 (0.77–2.25)
Iron, metal works, and foundries	16/502	0.75	0.75 (0.41–4.36)	31/829	0.89	0.91 (0.61–1.36)
Construction	13/440	0.65	0.66 (0.37–1.17)	26/774	0.78	0.80 (0.52–1.24)
Retail and wholesale trade	26/518	1.26	1.28 (0.83–1.98)	38/923	1.00	1.02 (0.70–1.49)
Transportation and storage	13/269	1.13	1.12 (0.63–2.01)	21/531	0.96	0.98 (0.61–1.56)
Accommodation and food service activities	< 5/64	–	–	7/153	1.16	1.17 (0.54–2.54)
Information and communication	8/132	1.48	1.46 (0.70–3.07)	8/212	0.94	0.92 (0.44–1.91)
Financial and insurance activities	< 5/105	–	–	5/162	0.75	0.74 (0.30–1.82)
Professional, scientific, and technical activities	11/122	2.33	2.29 (1.19–4.39)	17/263	1.65	1.61 (0.95–2.71)
Administrative and support service activities	< 5/80	–	–	6/265	0.53	0.52 (0.23–1.19)
Public administration and defense	20/494	0.99	0.97 (0.60–1.58)	41/928	1.12	1.10 (0.76–1.60)
Education	8/111	1.68	1.62 (0.77–3.42)	14/253	1.38	1.32 (0.74–2.34)
Human health and social work activities	< 5/119	–	–	8/218	0.90	0.87 (0.42–1.80)
Hospital and practitioner work	< 5/82	–	–	5/141	0.87	0.84 (0.34–2.08)
Job exposure matrices						
Benzene	12/385	0.74	0.74 (0.41–1.35)	24/714	0.79	0.80 (0.51–1.25)
Dichloromethane	21/521	1.00	1.00 (0.62–1.61)	35/894	0.96	0.98 (0.66–1.44)
Gasoline	6/243	0.57	0.57 (0.25–1.32)	13/432	0.70	0.72 (0.40–1.28)
Birth years > 1974^c^	5/199	0.61	0.61 (0.25–1.53)	10/340	0.69	0.72 (0.37–1.38)
Toluene	15/427	0.85	0.85 (0.49–1.47)	26/799	0.76	0.78 (0.50–1.20)
Birth years > 1974^c^	13/351	0.92	0.93 (0.51–1.67)	22/631	0.83	0.86 (0.53–1.38)
High/very high social contact	14/250	1.33	1.30 (0.74–2.30)	25/517	1.23	1.20 (0.77–1.87)

a Crude odds ratios

b Odds ratios adjusted for birth place (urban vs. rural/small town) and paternal age (continuous)

c Restricted to years when benzene was less commonly used

**Table 3:** Odds ratios (ORs) and 95% confidence intervals (CIs) for associations between paternal occupation/occupational exposures, offspring yolk sac tumors and teratomas, stratified by paternal exposure window, 1968–2016

Occupational exposure	Paternal exposure window					
	Three months preconception to birth			Birth to diagnosis		
	Case *n*/control *n*	OR^a^	aOR^b^ (95% CI)	Case *n*/control *n*	OR^a^	aOR^b^ (95% CI)
**Yolk sac tumors**	40/3,527			45/3,971		
Occupational industry						
Manufacturing	8/915	0.71	0.74 (0.33–1.64)	14/1,456	0.90	0.96 (0.50–1.85)
Iron, metal works, and foundries	< 5/502	–	–	7/829	0.80	0.83 (0.37–1.89)
Retail and wholesale trade	10/518	1.92	1.84 (0.87–3.91)	10/923	1.18	1.17 (0.55–2.38)
Public administration and defense	5/494	1.07	1.06 (0.40–2.80)	9/928	1.24	1.19 (0.56–2.54)
Job exposure matrices						
Benzene	< 5/385	–	–	6/714	0.93	0.93 (0.38–2.28)
Dichloromethane	5/521	0.87	0.87 (0.33–2.27)	7/894	0.86	0.88 (0.39–2.02)
Toluene	< 5/427	–	–	6/799	0.78	0.81 (0.33–1.95)
High/very high social contact	6/250	2.57	2.60 (1.04–6.47)	8/517	2.46	2.30 (1.02–5.18)
**Teratomas**	54/3,527			58/3,971		
Occupational industry						
Agriculture, forestry, fishing	< 5/120	–	–	6/220	1.82	1.99 (0.80–4.92)
Manufacturing	12/915	0.88	0.88 (0.36–1.71)	17/1,456	0.78	0.80 (0.44–1.44)
Food and beverage industry	5/155	2.41	2.40 (0.89–6.47)	6/320	1.51	1.57 (0.64–3.82)
Iron, metal works, foundries	5/502	0.72	0.73 (0.28–1.87)	8/829	0.66	0.68 (0.32–1.45)
Construction	7/440	0.97	1.05 (0.46–2.38)	12/774	1.15	1.23 (0.64–2.37)
Retail and wholesale trade	7/518	0.90	0.94 (0.42–2.13)	12/923	0.89	0.94 (0.49–1.81)
Transportation and storage	< 5/269	–	–	8/531	1.03	1.05 (0.49–2.28)
Public administration and defense	8/494	1.02	0.98 (0.45–2.12)	17/928	1.47	1.48 (0.82–2.69)
Job exposure matrices						
Benzene	< 5/385	–	–	5/714	0.47	0.48 (0.19–1.22)
Dichloromethane	6/521	0.80	0.80 (0.34–1.92)	7/894	0.49	0.51 (0.23–1.13)
Toluene	< 5/427	–	–	6/799	0.50	0.53 (0.22–1.25)
High/very high social contact	5/250	1.22	1.19 (0.45–3.13)	7/517	0.85	0.85 (0.37–1.93)

a Crude odds ratios

b Odds ratios adjusted for birth place (urban v. small towns/rural) and paternal age (continuous)

**Table 4:** Odds ratios (ORs) and 95% confidence intervals (CIs) for associations between maternal occupation/occupational exposures and offspring germ cell tumors, stratified by maternal exposure window, 1968–2016

Occupational exposure	Maternal exposure window					
	Conception to birth			Birth to diagnosis		
	Case *n*/control *n*	OR^a^	aOR^b^ (95% CI)	Case *n*/control *n*	OR^a^	aOR^b^ (95% CI)
Total	133/3,070			155/3,633		
Occupational industry						
Manufacturing	18/402	1.04	1.08 (0.64–1.82)	33/748	1.11	1.17 (0.78–1.76)
Textile, clothing, and leather industry	< 5/69	–	–	9/131	1.82	1.98 (0.97–4.05)
Iron, metal works, and foundries	9/127	1.69	1.74 (0.85–3.54)	18/298	1.60	1.68 (0.99–2.82)
Retail and wholesale trade	13/382	0.75	0.78 (0.43–1.40)	25/646	0.94	0.99 (0.63–1.54)
Transportation and storage	5/61	1.83	1.86 (0.73–4.78)	6/122	1.12	1.15 (0.50–2.67)
Accommodation and food service activities	< 5/68	–	–	6/207	0.69	0.73 (0.31–1.68)
Information and communication	< 5/84	–	–	7/163	0.99	1.00 (0.46–2.19)
Financial and insurance activities	8/151	1.23	1.22 (0.58–2.55)	10/206	1.16	1.15 (0.60–2.23)
Professional, scientific, and technical activities	< 5/78	–	–	6/172	0.85	0.85 (0.37–1.96)
Administrative and support service activities	< 5/54	–	–	6/208	0.73	0.75 (0.32–1.74)
Public administration and defense	43/1,002	1.03	1.00 (0.68–1.47)	80/1,925	1.01	1.01 (0.72–1.41)
Education	10/96	2.58	2.45 (1.23–4.90)	15/262	1.46	1.42 (0.82–2.49)
Human health and social work activities	35/601	1.40	1.39 (0.93–2.08)	44/944	1.16	1.14 (0.79–1.65)
Hospital and practitioner work	27/457	1.42	1.41 (0.91–2.19)	32/709	1.12	1.10 (0.74–1.65)
Daycares, kindergartens, and homes for children	< 5/62	–	–	6/129	1.10	1.10 (0.47–2.56)
Welfare institutions	8/86	1.99	2.01 (0.95–4.27)	12/211	1.44	1.43 (0.77–2.63)
Job exposure matrices						
Benzene	5/112	0.98	0.95 (0.38–2.38)	12/266	1.08	1.06 (0.58–1.95)
Dichloromethane	10/153	1.47	1.50 (0.77–2.95)	13/352	0.90	0.92 (0.51–1.65)
Gasoline	< 5/36	–	–	7/82	2.15	2.14 (0.96–4.76)
Birth years > 1974^c^	< 5/32	–	–	5/65	1.74	1.74 (0.69–4.40)
Toluene	5/95	1.22	1.21 (0.48–3.05)	12/244	1.21	1.23 (0.67–2.26)
Birth years > 1974^c^	5/79	1.43	1.40 (0.55–3.57)	9/189	1.10	1.11 (0.55–2.22)
High/very high social contact	16/246	1.57	1.52 (0.88–2.65)	27/560	1.20	1.19 (0.77–1.84)

a Crude odds ratios

b Odds ratios adjusted for birth place (urban vs. rural/small town) and maternal age (continuous)

c Restricted to years when benzene was less commonly used

**Table 5:** Odds ratios (ORs) and 95% confidence intervals (CIs) for associations between maternal occupation/occupational exposures, and offspring yolk sac tumors and teratomas, stratified by maternal exposure window, 1968–2016

Occupational exposure	Maternal exposure window					
	Conception to birth			Birth to diagnosis		
	Case *n*/control *n*	OR^a^	aOR^b^ (95% CI)	Case *n*/control *n*	OR^a^	aOR^b^ (95% CI)
**Yolk sac tumors**	39/3070			43/3633		
Occupational industry						
Manufacturing	< 5/402	–	–	7/748	0.92	0.92 (0.39–2.17)
Public administration and defense	13/1,002	1.07	1.14 (0.57–2.27)	18/1,925	0.96	1.01 (0.53–1.93)
Education	6/96	5.45	5.27 (1.94–14.28)	7/262	4.69	4.78 (1.90–12.01)
Human health and social work activities	11/601	1.68	1.55 (0.74–3.25)	14/944	1.81	1.78 (0.90–3.52)
Hospital and practitioner work	7/457	1.45	1.38 (0.58–3.26)	10/709	1.70	1.69 (0.80–3.57)
Job exposure matrices						
High/very high social contact	11/246	4.95	4.63 (2.08–10.37)	12/560	3.60	3.59 (1.69–7.62)
**Teratomas**	48/3,070			56/3,633		
Occupational industry						
Manufacturing	7/402	1.19	1.15 (0.50–2.64)	12/748	1.31	1.38 (0.70–2.71)
Retail and wholesale trade	< 5/382	–	–	10/646	1.00	1.08 (0.53–2.19)
Public administration and defense	16/1,002	1.10	1.02 (0.54–1.91)	20/1,925	1.21	1.22 (0.70–2.14)
Human health and social work activities	15/601	1.83	1.91 (1.00–3.63)	13/944	0.95	0.96 (0.50–1.82)
Hospital and practitioner work	11/457	1.61	1.62 (0.80–3.28)	9/709	0.80	0.81 (0.39–1.69)
Job exposure matrices						
Benzene	< 5/112	–	–	5/266	1.35	1.33 (0.52–3.43)
Dichloromethane	< 5/153	–	–	7/352	0.96	0.98 (0.38–2.53)
Toluene	< 5/95	–	–	5/244	2.12	2.10 (0.91–4.83)
High/very high social contact	< 5/246	–	–	5/560	0.55	0.55 (0.21–1.42)

a Crude odds ratios

b Odds ratios adjusted for birth place (urban vs. small towns/rural) and maternal age (continuous)

## Data Availability

As per the EU General Data Protection Regulation, data cannot at the present time be made available.
