# Supplementary material for: Parental occupation and childhood germ cell tumors: a case–control study in Denmark, 1968–2016
Source: Cancer Causes Control. Author manuscript; Available in PMC 2021 Aug 17. (PMC8236473; doi:10.1007/s10552-021-01434-0)
Supplement: 1713147_Supp_tab [file NIHMS1713147-supplement-1713147_Supp_tab.docx]

**Supplemental File 1.** Details of gestational age calculation.

From 1968-1972, no gestational age variable was collected; therefore, children born during this period were assigned the average gestational age in the Danish population (40 weeks or 280 days). For the period 1973-1977, we had access to a categorical gestational age variable imputed in weeks and collected by midwives. Children born between 1978-1996 also had gestational age recorded in weeks. To transform this into a continuous variable, we assigned each child the midpoint value of their gestational age category. Children born after 1997 had a gestational age value recorded in days. For the 3.9% of children who had missing gestational age, multiple imputation was performed using birthweight, birth length, placental weight, child’s sex, birth place, maternal smoking status, labor interventions or procedures, and presence of congenital malformations as predictors when available.

| **Supplementary Table 1.** Discordant case-control sets, adjusted odds ratios (aORs) and 95% confidence intervals (CIs) for associations between paternal occupation/occupational exposures and offspring germ cell tumors, stratified by paternal exposure window, 1968-2016.^a^ | | | | | | | | | | | | | | | |  |
| --- | --- | --- | --- | --- | --- | --- | --- | --- | --- | --- | --- | --- | --- | --- | --- | --- |
|  | |  | Paternal exposure window | | | | | | | | | | | | |  |
|  | |  | Three months preconception to birth | | | | |  | | Birth to diagnosis | | | | | |  |
| Occupational exposure | |  | Case exposed; control set unexposed |  | Case unexposed; control set exposed |  | aOR^b^ (95% CI) | |  | | Case exposed; control set unexposed |  | Case unexposed; control set exposed |  | aOR^b^ (95% CI) | |
| *Occupational industry* | | | | | | | | | | | | | | | |  |
| Agriculture, forestry, fishing | |  | - |  | - |  | - | |  | | 3 (1.8) |  | 95 (58.3) |  | 1.33 (0.70-2.53) | |
| Manufacturing | |  | 1 (0.7) |  | 112 (75.7) |  | 0.90 (0.61-1.33) | |  | | 0 (0.0) |  | 107 (65.6) |  | 0.93 (0.67-1.31) | |
|  | Food and beverage industry |  | 1 (0.7) |  | 82 (55.4) |  | 1.41 (0.70-2.85) | |  | | 1 (0.7) |  | 110 (67.5) |  | 1.31 (0.77-2.25) | |
|  | Iron, metal works, and foundries |  | 0 (0.0) |  | 122 (82.4) |  | 0.75 (0.41-4.36) | |  | | 0 (0.0) |  | 131 (80.4) |  | 0.91 (0.61-1.36) | |
| Construction | |  | 2 (1.4) |  | 126 (85.2) |  | 0.66 (0.37-1.17) | |  | | 0 (0.0) |  | 135 (82.8) |  | 0.80 (0.52-1.24) | |
| Retail and wholesale trade | |  | 0 (0.0) |  | 114 (77.0) |  | 1.28 (0.83-1.98) | |  | | 1 (0.6) |  | 121 (74.2) |  | 1.02 (0.70-1.49) | |
| Transportation and storage | |  | 1 (0.7) |  | 109 (73.7) |  | 1.12 (0.63-2.01) | |  | | 2 (1.2) |  | 137 (84.1) |  | 0.98 (0.61-1.56) | |
| Accommodation and food service activities | |  | - |  | - |  | - | |  | | 4 (2.5) |  | 90 (55.2) |  | 1.17 (0.54-2.54) | |
| Information and communication | |  | 4 (2.7) |  | 76 (51.4) |  | 1.46 (0.70-3.07) | |  | | 3 (1.8) |  | 103 (63.2) |  | 0.92 (0.44-1.91) | |
| Financial and insurance activities | |  | - |  | - |  | - | |  | | 2 (1.2) |  | 98 (60.1) |  | 0.74 (0.30-1.82) | |
| Professional, scientific, and technical activities | |  | 2 (1.4) |  | 67 (45.3) |  | 2.29 (1.19-4.39) | |  | | 1 (0.6) |  | 111 (68.1) |  | 1.61 (0.95-2.71) | |
| Administrative and support service activities | |  | - |  | - |  | - | |  | | 0 (0.0) |  | 108 (66.3) |  | 0.52 (0.23-1.19) | |
| Public administration and defense | |  | 1 (0.7) |  | 119 (80.4) |  | 0.97 (0.60-1.58) | |  | | 1 (0.6) |  | 121 (74.2) |  | 1.10 (0.76-1.60) | |
| Education | |  | 4 (2.7) |  | 67 (45.3) |  | 1.62 (0.77-3.42) | |  | | 5 (3.1) |  | 109 (66.9) |  | 1.32 (0.74-2.34) | |
| Human health and social work activities | |  | - |  | - |  | - | |  | | 3 (1.8) |  | 107 (65.6) |  | 0.87 (0.42-1.80) | |
|  | Hospital and practitioner work |  | - |  | - |  | - | |  | | 3 (1.8) |  | 81 (49.7) |  | 0.84 (0.34-2.08) | |
| *Job exposure matrices* | | | | | | | | | | | | | | | |  |
| Benzene | |  | 0 (0.0) |  | 117 (79.1) |  | 0.74 (0.41-1.35) | |  | | 0 (0.0) |  | 136 (83.4) |  | 0.80 (0.51-1.25) | |
| Dichloromethane | |  | 2 (1.4) |  | 119 (80.4) |  | 1.00 (0.62-1.61) | |  | | 1 (0.6) |  | 127 (77.9) |  | 0.98 (0.66-1.44) | |
| Gasoline | |  | 0 (0.0) |  | 101 (68.2) |  | 0.57 (0.25-1.32) | |  | | 0 (0.0) |  | 135 (82.8) |  | 0.72 (0.40-1.28) | |
|  | Birth years >1974^c^ |  | 0 (0.0) |  | 81 (68.1) |  | 0.61 (0.25-1.53) | |  | | 0 (0.0) |  | 110 (83.3) |  | 0.72 (0.37-1.38) | |
| Toluene | |  | 1 (0.7) |  | 123 (83.1) |  | 0.85 (0.49-1.47) | |  | | 0 (0.0) |  | 134 (82.2) |  | 0.78 (0.50-1.20) | |
|  | Birth years >1974^c^ |  | 1 (0.8) |  | 99 (83.2) |  | 0.93 (0.51-1.67) | |  | | 0 (0.0) |  | 107 (81.1) |  | 0.86 (0.53-1.38) | |
| High/very high social contact | |  | 3 (2.0) |  | 106 (71.6) |  | 1.30 (0.74-2.30) | |  | | 2 (1.2) |  | 127 (77.9) |  | 1.20 (0.77-1.87) | |
| ^a^Discordant sets and ORs not provided for exposures with <5 cases; discordant sets presented as, n (% of all case-control sets included for analysis). | | | | | | | | | | | | | | | |  |
| ^b^Odds ratios adjusted for birth place (urban v. rural/small town) and paternal age (continuous). | | | | | | | | | | | | | | | |  |
| ^c^Restricted to years when benzene was less commonly used. | | | | | | | | | | | | | | | |  |

| **Supplementary Table 2.** Discordant case-control sets, adjusted odds ratios (aORs) and 95% confidence intervals (CIs) for associations between maternal occupation/occupational exposures and offspring germ cell tumors, stratified by maternal exposure window, 1968-2016.^a^ | | | | | | | | | | | | | |
| --- | --- | --- | --- | --- | --- | --- | --- | --- | --- | --- | --- | --- | --- |
|  | |  | Maternal exposure window | | | | | | | | | | |
|  | |  | Conception to birth | | | | |  | Birth to diagnosis | | | | |
| Occupational exposure | |  | Case exposed; control set unexposed |  | Case unexposed; control set exposed |  | aOR^b^ (95% CI) |  | Case exposed; control set unexposed |  | Case unexposed; control set exposed |  | aOR^b^ (95% CI) |
| *Occupational industry* | | | | | | | | | | | | | |
| Manufacturing | |  | 2 (1.5) |  | 102 (76.7) |  | 1.08 (0.64-1.82) |  | 1 (0.7) |  | 115 (74.2) |  | 1.17 (0.78-1.76) |
|  | Textile, clothing, and leather industry |  | - |  | - |  | - |  | 5 (3.2) |  | 67 (43.2) |  | 1.98 (0.97-4.05) |
|  | Iron, metal works, and foundries |  | 4 (3.0) |  | 64 (48.1) |  | 1.74 (0.85-3.54) |  | 3 (1.9) |  | 100 (64.5) |  | 1.68 (0.99-2.82) |
| Retail and wholesale trade | |  | 2 (1.5) |  | 109 (82.0) |  | 0.78 (0.43-1.40) |  | 1 (0.7) |  | 121 (78.1) |  | 0.99 (0.63-1.54) |
| Transportation and storage | |  | 4 (3.0) |  | 39 (29.3) |  | 1.86 (0.73-4.78) |  | 4 (2.6) |  | 78 (50.3) |  | 1.15 (0.50-2.67) |
| Accommodation and food service activities | |  | - |  | - |  | - |  | 3 (1.9) |  | 91 (58.7) |  | 0.73 (0.31-1.68) |
| Information and communication | |  | - |  | - |  | - |  | 3 (1.9) |  | 88 (56.8) |  | 1.00 (0.46-2.19) |
| Financial and insurance activities | |  | 3 (2.3) |  | 70 (52.6) |  | 1.22 (0.58-2.55) |  | 1 (0.7) |  | 97 (62.6) |  | 1.15 (0.60-2.23) |
| Professional, scientific, and technical activities | |  | - |  | - |  | - |  | 2 (1.3) |  | 84 (54.2) |  | 0.85 (0.37-1.96) |
| Administrative and support service activities | |  | - |  | - |  | - |  | 2 (1.3) |  | 81 (52.3) |  | 0.75 (0.32-1.74) |
| Public administration and defense | |  | 0 (0.0) |  | 88 (66.2) |  | 1.00 (0.68-1.47) |  | 0 (0.0) |  | 75 (48.4) |  | 1.01 (0.72-1.41) |
| Education | |  | 5 (3.8) |  | 52 (39.1) |  | 2.45 (1.23-4.90) |  | 4 (2.6) |  | 99 (63.9) |  | 1.42 (0.82-2.49) |
| Human health and social work activities | |  | 0 (0.0) |  | 94 (70.7) |  | 1.39 (0.93-2.08) |  | 1 (0.7) |  | 110 (71.0) |  | 1.14 (0.79-1.65) |
|  | Hospital and practitioner work |  | 2 (1.5) |  | 97 (72.9) |  | 1.41 (0.91-2.19) |  | 1 (0.7) |  | 120 (77.4) |  | 1.10 (0.74-1.65) |
|  | Daycares, kindergartens, and homes for children |  | - |  | - |  | - |  | 3 (1.9) |  | 70 (45.2) |  | 1.10 (0.47-2.56) |
|  | Welfare institutions |  | 5 (3.8) |  | 56 (42.1) |  | 2.01 (0.95-4.27) |  | 5 (3.2) |  | 93 (60.0) |  | 1.43 (0.77-2.63) |
| *Job exposure matrices* | | | | | | | | | | | | | |
| Benzene | |  | 2 (2.3) |  | 66 (49.6) |  | 0.95 (0.38-2.38) |  | 2 (1.3) |  | 112 (72.3) |  | 1.06 (0.58-1.95) |
| Dichloromethane | |  | 3 (2.3) |  | 73 (54.9) |  | 1.50 (0.77-2.95) |  | 2 (1.3) |  | 115 (74.2) |  | 0.92 (0.51-1.65) |
| Gasoline | |  | - |  | - |  | - |  | 5 (3.2) |  | 55 (35.5) |  | 2.14 (0.96-4.76) |
|  | Birth years >1974^c^ |  | - |  | - |  | - |  | 4 (3.1) |  | 51 (38.9) |  | 1.74 (0.69-4.40) |
| Toluene | |  | 2 (1.5) |  | 55 (41.4) |  | 1.21 (0.48-3.05) |  | 2 (1.3) |  | 98 (63.2) |  | 1.23 (0.67-2.26) |
|  | Birth years >1974^c^ |  | 2 (1.8) |  | 48 (42.1) |  | 1.40 (0.55-3.57) |  | 2 (1.5) |  | 84 (64.1) |  | 1.11 (0.55-2.22) |
| High/very high social contact | |  | 3 (2.3) |  | 84 (63.2) |  | 1.52 (0.88-2.65) |  | 2 (1.3) |  | 115 (74.2) |  | 1.19 (0.77-1.84) |
| ^a^Discordant sets and ORs not provided for exposures with <5 cases; discordant sets presented as, n (% of all case-control sets included for analysis). | | | | | | | | | | | | | |
| ^b^Odds ratios adjusted for birth place (urban v. rural/small town) and maternal age (continuous). | | | | | | | | | | | | | |
| ^c^Restricted to years when benzene was less commonly used. | | | | | | | | | | | | | |
